# Possible Causes of Malnutrition in Melghat, a Tribal Region of Maharashtra, India

**DOI:** 10.5539/gjhs.v6n5p164

**Published:** 2014-05-30

**Authors:** Tannaz J. Birdi, Sujay Joshi, Shrati Kotian, Shimoni Shah

**Affiliations:** 1 The Foundation for Medical Research, Mumbai, India

**Keywords:** diet diversity, Melghat, malnutrition, micronutrient, weaning, kitchen garden (KG), green leafy vegetables (GLVs), Public Distribution System (PDS), Integrated Child Development Scheme (ICDS), Anganwadi (AW), households (HHs), Participatory Action Research (PAR)

## Abstract

Melghat, situated in Amravati District of Maharashtra, India is a tribal region with amongst the highest numbers of malnutrition cases. This paper focuses on possible causes of malnutrition in the Dharni block of Melghat. Quantitative survey recorded the existing burden of malnutrition, kitchen garden (KG) practices, Public Distribution System, food provisioning, Anganwadi services and hygiene/sanitation in the community. Additionally a qualitative study was undertaken to understand the community’s perspective on nutrition, cultural beliefs, spending habits and other factors contributing to malnutrition. Malnutrition was found to be highly prevalent amongst all age groups with 54% children aged 1-5 years and 43% adults aged ≥ 20 years being severe to moderately underweight. A major cause for malnutrition in children was faulty child care practices. Data on food provisioning revealed that while the caloric needs of the community were substantially met by consumption of cereals and pulses, minimal consumption of green leafy vegetables (GLVs) could lead to micronutrient deficiency in the community. KGs, which provide GLVs, were mainly cultivated in monsoon (98%) which declined to merely 4% in summer. The benefits of government schemes though targeted at malnourished children were often shared by the entire household and thus got diluted. Key finding was that nutrition interventions should be designed to address the entire household and emphasis should be given to appropriate nutrition education, without which distributing food or increasing income would have minimal effect.

## 1. Introduction

Malnutrition is the underlying cause of 3.5 million deaths and 35% of disease burden in children below 5 years of age (Black et al., 2008). Besides its immediate short term consequences, long term effects have profound implication on children’s growth and cognitive abilities which may translate into reduced economic development of future generations (Sandjaja et al., 2013; Hoddinott et al., 2013). Additionally malnutrition in pregnant women leads to poor fetal growth leading to infants with low birth weight. Due to its far reaching consequences major development agencies have addressed this problem. Copenhagen Consensus 2008 concluded nutrition interventions to be amongst the most cost effective in the development programme (Gillespie, Haddad, Mannar, Menon, & Nisbett, 2013). The ‘Scaling Up Nutrition’ movement initiated in 2010 represents the growing interest in nutrition globally (Gillespie et al., 2013).

Reduction of malnutrition is a multisectoral activity adding complexity to its implementation. In India despite economic growth, nutritional benefits have been limited (Subramanyam, Kawachi, Berkman, & Subramanian, 2011) thus generating a growing consensus amongst stakeholders on need for multisectoral reform (Swaminathan, Edward, & Kurpad, 2013) leading to the Right to Food initiative (Mander, 2012). Some existing government schemes targeting nutrition in India are Public Distribution System (PDS), Integrated Child Development Scheme (ICDS), Mid-Day Meal and Anganwadi (AW) scheme. These schemes target hunger and not nutrition, with food quality often being poor and quantity insufficient (Von Braun, Ruel, & Gulati, 2008). Although these have potential, they need to be better integrated and coordinated to increase efficiency and effectiveness.

Melghat, comprising of two blocks, Dharni and Chikhaldhara, situated in Amravati district is home to the tiger reserve of Maharashtra. It’s a hilly area spreading across 320 villages comprising mainly of Korku tribe. Dharni’s population of 147 086 persons has a sex ratio of 962 females per 1000 males (http://censusindia.gov.in/PopulationFinder/Sub_Districts_Master.aspx?state_code=27&district_code=07).

This region now has amongst the highest numbers of malnutrition and infant mortality cases (R. Singh & P. Singh, 2008). By governments own admission, under 5 mortality rate is 70 per 1000 live births (http://www.maha-arogya.gov.in/Undernutrition/Absolutely%20Final%20report%20Undernutrition%20492012.pdf). This paper attempts to estimate the burden of malnutrition in Melghat as well as understand the factors contributing to it. It broadly aims to highlight the availability of food items, type of daily diet consumed by families, income and spending habits of the community and also childcare practices that throws light on malnutrition amongst children.

## 2. Method

### 2.1 Selection of Villages

**Figure 1:**
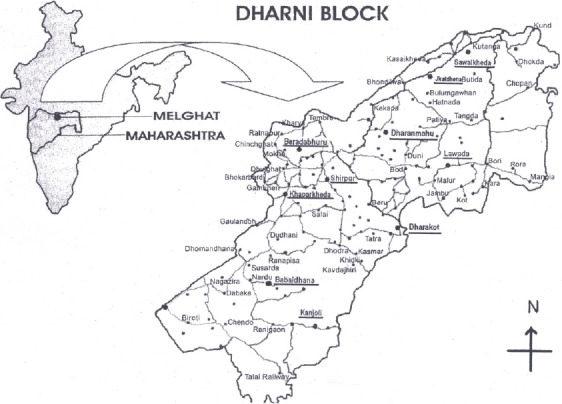
Representative map of the study area

Amongst the 152 villages in Dharni block, 10 were randomly selected (Figure 1) based on geographic location to ensure wide distribution within the block.

### 2.2 Selection of Households (HHs)

List of all HHs that had atleast one child aged <6 years was obtained from the AW. These HHs were invited to a community meeting as well as approached individually to explain the nature of the study. A total of 396 HHs were then included based on inclusion criteria and voluntary participation.

### 2.3 Malnutrition Classification

Height and weight of family members, at the time of interview were recorded to determine their malnutrition grade. Members aged 1-5 years were classified as severe, moderate, mild underweight and normal weight based on the weight for age Z score estimated using nutritional survey in the WHO-Anthro tool (WHO, 2009). Members aged > 5 - 19 years were classified as underweight and normal based on BMI percentile classification, estimated using BMI calculator by CDC (2010). Members aged > 20 years were classified as severe, moderate, mild thinness and normal weight based on WHO standard BMI cutoff’s (2004).

### 2.4 Quantitative Data Collection

Any one adult member (> 21 years) that was available in the HH at the time of interview was interviewed. Quantitative data on demographics, income sources, migration, existing kitchen garden (KG) practices, PDS, child care practices, AW and immunization services, water hygiene and sanitation was collected by means of a structured questionnaire. Dietary intake patterns and food provisioning habits of HHs were evaluated to obtain diet diversity and quantity of food intake.

### 2.5 Qualitative Data Collection

Participatory Action Research (PAR) activities and meetings were conducted with community groups and school children to get a wider perspective on their nutrition and childcare practices and substantiate the information gathered through quantitative survey. Data was also obtained by informal interviews of 100 HHs across the intervention villages using a semi structured questionnaire. Qualitative data was also obtained to deduce the percentage of income spent during weekly markets and of that how much was spent on food items in order to estimate food purchasing power of a Melghat HH.

### 2.6 Statistical Analysis

Quantitative data was entered in CSPro 4.1 and imported into SPSS v19 for further analysis. Qualitative interviews of each HH were considered as a respondent and were audio recorded and translated into English. Saturation levels for responses were achieved and data was analyzed using ATLAS ti7.

### 2.7 Ethical Consideration

Clearance was obtained from the Institutions’ Ethics Committee (Institute/IEC/KG/01/2012).

## 3. Results

### 3.1 Family Demographics

Table 1 shows gender ratio within various age groups. Average HH comprised of 7 members with 20% HHs having ≥ 4 children aged 0-13 years. No gender bias was found for preference at birth, weight amongst children or for literacy in 6-19 year olds. However, in older age group (≥ 20 years) 50% females were illiterate as opposed to 22% males. PAR activities highlighted gender bias in terms of amount and type of work carried out. While women performed HH and agricultural tasks, men carried out agricultural tasks, animal rearing and firewood collection along with having financial control. It was also observed that women even till their last month of pregnancy were involved in heavy work like bringing water from open well however, no official data was documented.

**Table 1:** Age and gender distribution in the study population

Age	Total in age group No. (%)	Gender per age group	
		Male No. (%)	Female No. (%)
0-1 year	214 (8)	94 (44)	120 (56)
>1-5 years	498 (18)	228 (46)	270 (54)
>5-10 years	248 (9)	95 (38)	153 (62)
11-19 years	314 (11)	147 (47)	167 (53)
20-45 years	1120 (41)	566 (51)	554 (49)
>45 years	343 (13)	175 (51)	168 (49)
Total	2737 (100)	1305 (48)	1432 (52)

### 3.2 Prevalence of Malnutrition

Malnutrition was not gender biased but children were most affected with 54% aged 1-5 years and 67% aged >5–10 years being severe to moderately underweight. Amongst others, 54% adolescents (11–19 yrs), 18% adults in the reproductive age group (20–45 years) and 25% adults > 45 years were found to be severe to moderately underweight. The higher level of malnutrition amongst boys aged 11-19 years was probably due to adolescent growth spurt. Amongst women, 20% in the reproductive age group were severe to moderately thin which is crucial in terms of its impact on pregnancy and child care (Table 2).

**Table 2:** Prevalence of Malnutrition in the study population

Age categories (years)	Malnutrition Grades	Gender		Total (%)
		Male (%)	Female (%)	
1 -5	Severe underweight	27.9	24.5	26.1
	Moderate underweight	30.8	25.3	27.9
	Mild underweight	28.8	29.6	29.3
	Normal weight	12.5	20.6	16.8
	Total	100.0	100.0	100.0

>5 -10	Underweight	60.5	71.8	67.5
	Normal	39.5	28.2	32.5
	Total	100.0	100.0	100.0

11 -19	Underweight	73.6	41.2	53.6
	Normal	26.4	58.8	46.4
	Total	100.0	100.0	100.0

20 – 45	Severe thinness	7.2	8.9	8.3
	Moderate thinness	7.7	11.2	9.9
	Mild thinness	28.4	27.8	28.0
	Normal weight	56.8	52.1	53.8
	Total	100.0	100.0	100.0

> 45	Severe thinness	16.5	10.1	13.1
	Moderate thinness	11.4	12.4	11.9
	Mild thinness	25.3	22.5	23.8
	Normal weight	46.9	55.1	51.2
	Total	100.0	100.0	100.0

Total	41.0	59.0	100.0

### 3.3 Perception of Malnutrition in the Community

Malnutrition was commonly perceived as extreme muscle wasting (28 respondents) followed by bulging stomach and skinny limbs (5 respondents), falling ill frequently (8 respondents) and anemia (2 respondents). Some reasons stated for malnutrition were early age of marriage (13 respondents), illness (8 respondents), less spacing between two children (6 respondents), and poor child care (23 respondents). Importance of child spacing was only with respect to availability of breast milk. Two superstitions listed as reasons for malnutrition were, child being cleaned with a *sari* of a woman other than the mother and a pregnant woman lifting water container of another woman in the village.

While 46 respondents did not have any knowledge on treating malnutrition, some responded taking their child to the hospital, although being unaware of the treatment (14 respondents). Some mentioned that treatment was given at the AW where the child received eggs, bananas and jaggery *chikki* (24 respondents). There were 3 respondents that preferred being treated by traditional healers rather than modern doctors.

Based on the responses obtained above it was concluded that understanding of malnutrition was poor in the community and was perceived to be a problem amongst children and not adults. No mention of micronutrient deficiency shows the lack of knowledge on its impact on malnutrition.

### 3.4 Nutrition Knowledge and Food Provisioning

Majority respondents when asked what constitutes a health daily diet responded that they consumed ‘*chapatti*’ (flat bread), pulse and rice daily. Accessibility and affordability of vegetables emerged as common reasons for poor nutrition (80 respondents). Frequency of market visits ranged from weekly to monthly while spending and price of produce were governed by seasonal fluctuations.

Average amount spent by a HH at weekly market was USD 6.75, half of which was spent on basic necessities and remaining on food. Tubers (onion and potato) were preferred followed by vegetables (brinjal and tomato). Green leafy vegetables (GLVs) were mainly consumed in winter. Fruits and meat as well as additional variety of food were purchased only on availability of extra money. Winter being the harvest season showed highest food availability and economic stability and this declined with the onset of summer.

Cereals and pulses were perennially consumed. Cereals were obtained not only from PDS (76%) but also from their farms (68%) and market (53%), suggesting insufficient and irregular PDS supply. Pulses were either bought from the market (74%) or obtained from their farm (67%). GLV’s were more commonly obtained from KGs along with other vegetables which were also supplemented from the market (94%). Respondents purchased minimal quantity of vegetables including GLVs due to lack of storage facility (15 respondents). Vegetable consumption was not considered important but the community did mention that it increased energy (23 respondents). One common reply given for all food categories was it improved blood (19 respondents). Traditional beliefs governed the consumption habit with vegetables including GLVs not being consumed for 4-5 months (summer and early monsoon). Qualitative data suggested that GLVs and other vegetables were consumed for variation in taste and not their nutritional value.

Fruits were mainly purchased for children as respondents were aware of their benefits. Some benefits listed were increased energy (14 respondents) and weight (3 respondents) and healthier body (62 respondents). Milk was expensive and it’s availability poor. Reasons for milk consumption were; gave strength (26 respondents) and suppressed hunger (3 respondents). PDS supplied oil and sugar only during festive occasions.

‘*Zeelu*’ (non-vegetarian food) was extremely popular in the community however, quantity consumed was minimal. Some of the benefits listed were improved health (31 respondents), increased weight (6 respondents) and improved food palatability.

### 3.5 Diet Diversification and Food Consumption

HHs were asked about the weekly purchase of different types of food groups and their quantity. Weekly provisioning data revealed that almost all HHs consumed cereals (wheat and rice), pulses (red gram *and* green gram), tubers (potato and onion) and other vegetables (brinjal and tomato) during winter and summer (Table 3). Additional varieties of other vegetables (lady’s finger and pumpkin) were consumed by 50% HHs during winter and only 22% HH during summer (Table 4). Consumption of additional cereal like *jowar* (*Sorghum vulgare*) reduced from 79% during winter to 47% during summer (Table 4). GLVs (spinach, fenugreek and cabbage) which were consumed by 80% HHs during winter decreased to 37% during summer (Table 3). Also as seen from table 4, the percent of HHs that consumed more than 2 types of GLVs reduced from 65% in winter to 19% in summer. Fruits were consumed by 87% and 76% HHs during winter and summer respectively (Table 3) with majority consuming only 1 variety mainly banana as it was cheap and readily available perennially as seen in table 4. Data reveals consumption of all food categories by almost all HHs during both seasons; however the variety reduced during summer.

**Table 3:** Seasonal comparison of diet and average amount of food consumed per person in households (HHs)

		Winter		Summer	

Food Category	Recommended dietary allowances for moderate activity* (gms)	Avg. quantity consumed/person/day¶ (gms)	% HHs that consumed the food item (%)	Avg. quantity consumed/person/day¶ (gms)	% HHs that consumed the food item (%)
Total Cereals	390	487.3	100	524.8	100
Pulses	82.5	88.2	99	93.5	99
GLV’s	100	33.6	80	15.1	37
Roots	200	46.8	97	44.6	93
Other Vegetables	200	53.0	99	55.9	97
Fish	-	14.8	63	17.5	47
Meat	-	20.2	87	18.9	80
Sugar	30	33.2	95	37.9	92
Fruits	100		87		76
Eggs	-		3		8

* Obtained from second edition of National Institute of Nutrition, 2010. Quantities averaged for male and female for comparison purpose. Moderate activity includes walking briskly (about 3½ miles per hour), climbing, gardening/yard work, dancing, walking short distances for fetching milk and vegetables, bicycling (less than 10 miles per hour), and weight training (a general light workout), yoga and pranayama, playing with children.

¶ Quantities averaged for HHs that consumed the particular food category.

**Table 4:** Seasonal comparison of diet diversity amongst the HHs

Food item	Variety	Winter	Summer

		HHs that consumed (%)	HHs that consumed (%)
Cereal	3-4 types	79	47
Pulse	≥ 2 types	72	83
GLV’s	≥ 2 types	65	19
Roots	≥ 2 types	57	43
Other Vegetables	3-5 types	50	22
Fruits	1 type	68	44

Meat was consumed by most HHs during winter (87%) as well as summer (80%) (Table 3). As it was expensive, average quantity purchased by a HH was ½ kg/month which was divided amongst all HH members. Fish was consumed by 63% HHs during winter and 47% during summer. Milk consumption was poor although 88% HHs owned cattle. Even amongst HHs that owned poultry (35%), egg consumption was low in both seasons mainly due to their preference for raising chickens for sale (70%). Hence, although livestock rearing was common (71%), it was not for HH consumption.

Table 3 also compares recommended dietary allowance per person/day with the amount consumed during winter and summer. Daily consumption per person was derived from weekly quantity purchased and divided by number of HH members above 5 years. It was averaged only for HHs that consumed the particular food category. Although cereals and pulses were sufficiently consumed, amount of all other food categories were lower, even more so during summer. Fruits, seafood, meat and poultry were consumed on rare occasions and mostly during the weekly market.

Data confirmed inadequate quantity of consumption and lack in diet diversity.

### 3.6 Homestead KG Practice

KG’s which are traditionally practiced were cultivated by 79% HHs mainly during monsoon (98%). KG cultivation declined to 33% in winter and to 4% in summer. Common crops cultivated were pumpkin (64% HHs) during monsoon and fenugreek (24% HHs), spinach (20% HHs), coriander (20% HHs) during winter.

### 3.7 Source of Income

With 78% HHs either owning or leasing land the major source of livelihood was agriculture and selling of cash crops. Rain fed or subsistence farming was practiced (73%) due to water shortage. Due to ground water sources drying up, only 14% cultivated summer crops even though 50% HHs had irrigation facilities.. Major crops cultivated were *tur* (81%), *jowar* (78%), soyabean (75%) and rice (72%). Respondents were mainly marginal (24%) farmers who owned <2.5 acres and small farmers (50%) who owned 2.5 to 5 acres of land. Majority HHs (96%) had some form of additional income like bamboo crafts, honey collection, sale of tobacco leaves and mill work. Economic stability being highest in winter decreased gradually during monsoons due to investments in farming activities. Seventy percent HHs had men migrating to nearby cities as laborers leaving women to attend to field and house work.

### 3.8 Spending Habits

Expenditure of a HH was important to note since it provided information on the communities spending habits in proportion to their income and the priority given to nutrition. Community had very little savings with daily expenses being taken care of by agricultural labour. Monthly ration was obtained at a subsidized rate of approximately USD 1.40 per person from the PDS. Minimal amount was spent on food other than cereals and pulses.

Preference to spend rather than save came out in qualitative findings. End of each harvest season saw a significant amount being spent on festivals. Major proportion of income was used to repay agriculture related loans (varying from USD 50 to 670) that were mainly obtained from local money lenders by pledging jewellery (67 respondents). Bank loans were perceived to be complicated and cumbersome. Those with a bank account used it mainly to receive money from various government schemes (49 respondents). Gold and silver jewellery was purchased as means of security to obtain loans in future. Income was also spent on electronics like televisions and mobiles (14 respondents), cosmetics, house repairs (16 respondents) and purchasing seeds (8 respondents).

### 3.9 Water Hygiene and Sanitation

Region showed poor water hygiene and sanitation practices. Drinking water was obtained either from public tap, open well or hand pump with majority (97% HHs) not treating it. Only 36% HHs placed the drinking water container on an elevated platform with almost all HHs (92%) covering it but not using a ladle/tap (93%). Water from well (61%) or lake/river (26%) was consumed while working outdoors. Inbuilt toilets were only present in 4% HHs as majority used open spaces. Washing hands after defecation (69%), cleaning child’s stool (37%), before and after eating (40% and 3% respectively), before cooking (71%) and after work (7%) was carried out poorly.

### 3.10 Child Care

#### 3.10.1 Breast Feeding and Complimentary Feeding Practices

Faulty practices were observed in the region. Colostrum and pre lacteal food was fed by 82% and only 19% mothers respectively. Most mothers (48%) stopped breast feeding at 24 months. Untreated water given to children pre-disposed them to infections. Mean age of initiating complimentary feeding was 9.5 months with 33% starting at only 1 year. Frequency of complementary feeding was <3 times/day amongst 64% respondents with most common foods being rice*/*pulse (81%), biscuits (72%), wheat/jowar *chappati* (34%) and local variety of junk food (29%). When mothers went to work, infants were often left behind with elders and/or siblings at home (34%).

Qualitative findings showed that chapattis given to children were thick making them inedible which was often perceived as the child not being ready for complimentary feeding, leading to delayed weaning. Infants left at home were often fed biscuits or junk food by elder/siblings as they were easier to feed. Poor quality snacks were commonly consumed (93 respondents) and pocket money ranging from USD 0.04–0.58 day was given to purchase it. Most respondents were aware that junk food consumption was unhealthy (72 respondents) while some respondents were unaware (17 respondents) and others believed they were nutritious (4 respondents).

#### 3.10.2 Immunization

Infants accompanying parents on field or migration missed the monthly vaccinations held at the AWs. Most respondents (93%) did not have an immunization card and amongst those that did, only 11 were complete.

#### 3.10.3 Deworming

During past 6 months, majority HHs (47%) had none of their children dewormed whereas 27% HHs had all children dewormed.

#### 3.10.4 Anganwadi Services

AWs, part of the ICDS program, provide supplementary food to children aged 0-6 years, pregnant women and breastfeeding mothers. Only 58% women availed their services at > 4 months of pregnancy and 8% did not avail any services. Some respondents (13%) were either unaware of services or some chose not to avail them (7%). Many respondents complained about the minimal quantity, poor quality and lack of variation in the food provided. ‘Spot-feeding’ which is mandatory was only observed amongst 12% HHs with majority (62%) carrying food back home to share with siblings and other HH members. Some AW workers expected mothers to sit at the AW and feed their child, thus preventing the mothers from availing their services due to feasibility.

#### 3.10.5 Schemes for Malnourished Children

ICDS has started Village Child Development Centers in AWs to provide special diet comprising of eggs and bananas, six times/day for one month, to children suffering from severe acute malnutrition. As per guidelines only 10-15 children in rural areas and 20-25 children in tribal areas would be eligible from an AW of 100 children. This scheme ensured that the child was no longer malnourished only temporarily and as no clear guidelines for funding is established it has resulted in the scheme being irregular and poorly implemented.

Some other government schemes are ***Khawati karja*** which provides wheat, rice, tur daal, oil, salt and spices to HH with a malnourished child. Besides the food being consumed by all HH members, this scheme lacks clarity on the eligibility criteria resulting in many eligible families not benefiting from it. In 2013, one time **monetary support** of USD 50 was given to families of malnourished children for their nutrition which was often spent on miscellaneous items.

Figure caption-Hard copy of Dharni Map collected in May 2009 from “Executive Magistrate Office”, Dharni and edited using coral draw for representation shows the 10 villages (underlined) selected for the study. Map not to scale.

## 4. Discussion

Both, government and voluntary sector in the last decade have introduced various schemes and poured large amount of money into Melghat to tackle malnutrition. However, malnutrition still exists amongst all age groups and therefore, the key question to be addressed is why is it still rampant.

Whilst the often stated reasons like; young age of marriage (Raj et al., 2010), insufficient spacing between births (Rutstein, 2005), large family size (Pelto et al., 1991), frequent episodes of diarrhea (Avachat, V. D. Phalke, D. B. Phalke, Aarif, & Kalakoti, 2011) and other illnesses still hold true, the present study also highlights other factors in the region that are directly associated with malnutrition.

As soon as Melghat was declared a reserved forest the Korkus were forced into a settled form of existence and their access to natural nutritional and medicinal plants was prohibited. This explains their poor knowledge on agriculture since historically there were hunters and gatherers. Additionally, extreme weather and geographical conditions along with water scarcity allows only rain fed subsistence farming. This often sees male members of family migrating for extended period to generate alternative source of income. Educating the community on agriculture and water harvesting techniques would help improve agricultural yield, aiding in economic and nutritional stability.

As the data suggests, only severe muscle wasting was considered as malnutrition. Although the community could list some signs, causes and treatment of malnutrition, none of them associated it to lack of a healthy balanced diet, thereby highlighting the central role of nutrition education in preventing malnutrition.

Lack in diet diversity was attributed to accessibility and affordability. Weekly markets did not ensure adequate purchase of vegetables even during winter due to lack of storage facilities. Furthermore the quantity purchased is usually insufficient for the entire HH resulting in inadequate nutrition. Affordability however was contraindicative based on their spending habits which highlight the community’s lack of preference to spend on food. This could be attributed to their inability to differentiate between alleviation of hunger and adequate nutrition. These observations emphasize the need for sustained nutrition education as increasing income alone will not improve nutritional levels. A Meta analysis undertaken by Ruel and Alderman (2013) to link income growth and anthropometrical measurements of mother and child showed that in some countries including India, nutrition indicators were poorer in comparison to their income.

Caloric requirement of a moderately working male is approximately 2800 Kcal and for females it ranges between 2220 Kcal to 2800 Kcal (Gopalan, Sastri, & Balasubramanian, 2004). Weekly provisioning data estimated that cereals and pulses provided approximately 2000 Kcal and since the consumption of other food categories was minimal, a chronic shortfall in caloric intake existed.

Improper breast feeding and complimentary feeding which have also been previously reported (Deshpande, Zodpey, & Vasudeo, 1996) seem to play a role in malnutrition amongst children. Educating mothers on importance of nutrition and providing methods to prepare healthy and easy to cook snacks could address the problem significantly.

Anganwadi’s are directly associated with providing nutrition and health to children, pregnant women and mothers. Our data suggests gaps in the services provided which can be strengthened. Other than food distribution, immunization and deworming need attention. It has been reported that micronutrient enrichment of school meals did not decrease anemia possibly because of the worm load (Osei et al., 2010). Thus AWs need to ensure adequate deworming along with providing nutritious mid day meal.

Generational nutritional deficiency sees an undernourished female giving birth to an undernourished child who then grows up to be an undernourished adult. In order to break this cycle, the government initiated the PDS scheme. However the PDS scheme, initiated by the government contributes only to food security and not ‘nutritional security’. Schemes targeting malnourished children have been diluted at HH level thereby not significantly benefiting the nutritional status of the concerned child. It is therefore important to design interventions that are HH centric and not child centric. Numerous schemes have also resulted in the community’s dependence on government for all development related issues.

In conclusion, the key learning of this study was demonstrating the need to empower the community to undertake self help measures rather than passively being dependant on handouts. The importance of sustained and appropriate nutrition and agriculture related education at all stages in achieving this cannot be overemphasized.
